# Ex Vivo High-Frequency Ultrasound of the Skin in the Morphologic Assessment of Basal Cell Carcinoma: A Comparative Study

**DOI:** 10.3390/diagnostics16183060

**Published:** 2026-09-21

**Authors:** Valentin-Tudor Popa, Bianca Natarâș, Sorina Tăban, Sorin Ursoniu, Alis Dema

**Affiliations:** 1 Anapatmol Research Centre, “Victor Babeș” University of Medicine and Pharmacy, Eftimie Murgu Square No. 2, 300041 Timișoara, Romania; popa.valentin@umft.ro (V.-T.P.);; 2 Doctoral School, “Victor Babeș” University of Medicine and Pharmacy, Eftimie Murgu Square No. 2, 300041 Timișoara, Romania; 3 Department of Functional Sciences III, Discipline of Public Health and History of Medicine, “Victor Babeș” University of Medicine and Pharmacy, 300041 Timișoara, Romania; 4 Centre for Translational Research and Systems Medicine, “Victor Babeș” University of Medicine and Pharmacy, Eftimie Murgu Square No. 2, 300041 Timișoara, Romania

**Keywords:** high-frequency ultrasound, ex vivo ultrasound, dermatology, basal cell carcinoma, method-comparison study

## Abstract

**Background/Objectives**: Noninvasive imaging of the skin in the assessment of basal cell carcinoma (BCC) is a promising field of research. Ultrasound stands out because of its ease of use, low price point and versatility. Ex vivo ultrasound can be used to obtain detailed measurements of tumor size, depth and margin assessment. These measurements can be compared with histopathological measurements. **Methods**: We performed a prospective study on formaldehyde-fixed tissue samples at the Pathology Department of Timisoara County Hospital. A total of 19 consecutive basal cell carcinomas from 17 patients were included in the study. **Results**: The study cohort was predominantly male (76.5% versus 23.5%), with a median age of 73 years (interquartile range: 62–77 years). Most lesions were excised from the head and neck region (84.2%) and were primarily classified as nodular (42.1%) and infiltrative (36.8%) subtypes by histopathological assessment. Bland–Altman analysis comparing ultrasound measurements to histopathology revealed no statistically significant systematic or proportional bias for tumor depth and deep margin measurements; however, the limits of agreement were wide for all three parameters: tumor depth (−3.35 to +3.77 mm), deep margin (−3.36 to +2.65 mm), and maximum diameter (−17.32 to 14.55 mm). Statistically significant proportional bias was observed in ultrasound-based assessments of tumor size. An additional exploratory analysis evaluated the diagnostic accuracy of ex vivo ultrasound in identifying tumors with invasion depths exceeding 2 mm relative to histological analysis. **Conclusions**: Our results suggest that ex vivo HFUS at 22 MHz is not interchangeable with histopathology. Nevertheless, the results reported in this pilot study will be useful to inform future research and will serve as a framework for reporting HFUS results in dermato-oncology.

## 1. Introduction

Noninvasive imaging of the skin in the assessment of BCC is a promising field of research. Emerging technologies such as confocal microscopy, optical coherence tomography (OCT) and high-frequency ultrasound of the skin (HFUS) are mentioned in the most recent guidelines for the diagnosis and treatment of BCC [1,2]. In vivo assessments can then be further correlated with histopathology and used to define better recommendations for both surgical and conservative management of this very frequent pathology [3,4]. Ultrasound stands out because of its ease of use, low price point and versatility in both clinical and surgical settings. A recent study by Crisan et al. presented a practical case series of both in vivo and ex vivo applications of HFUS in the management of BCC: assessment of tumor infiltration depth, lateral margin assessment, tumor mapping and evaluation of operability, selection of cases for Mohs surgery and ex vivo tumor margin assessment [5]. Precise HFUS measurements could also help select lesions that are candidates for topical therapy or provide an objective measurement of treatment response [6,7,8].

There is no consensus on how to use quantitative measurements in the management of basal cell carcinoma. Rimbert et al. surveyed 225 pathologists in France on the practice of systematically measuring microscopic margins for BCC [9]. Almost one in five (19.6%) of the respondents reported that they measure microscopic margins only in certain cases. Specialized dermatopathologists more frequently did not measure BCC and preferred a qualitative approach using terms such as “close limit to the tumor”. The main reasons for not measuring excision margins were the lack of clear recommendations and the possibility of tissue distortion. A recent guideline developed under the coordination of the European Association of Dermato-Oncology makes no mention of precise histopathological measurements, suggesting only that the status of the lateral and deep margins be reported [10]. A recent national German guideline explicitly recommends mentioning vertical tumor diameter (tumor thickness) in the pathology report and uses horizontal tumor diameter together with location for risk stratification [2]. Tumors that are less than 2 millimeters in vertical thickness and present a low recurrence risk are eligible for local treatment, although margin-controlled excision is still considered the first-line therapy whenever feasible. Ex vivo HFUS can be used to measure both horizontal and vertical tumor diameter.

Ex vivo ultrasound can offer both quantitative and qualitative measurements. The use of HFUS on ex vivo surgical specimens of BCC was pioneered in 2016 by Pasquali et al. and showed promising results [11]. Wang et al. explored the use of ultrasound in evaluating histologic subtypes of BCC based on morphology [12]. Popescu et al. measured in vivo tumor depth in BCC and used a Bland–Altman analysis to measure agreement between ultrasound measurements and histopathology. They reported clinically acceptable agreement with histopathology when measuring in vivo tumor depth [13]. Using ultrasound to measure tumors ex vivo can avoid errors posed by tissue shrinkage after biopsy. Dauendorffer et al. explored the problem of tissue shrinkage in cutaneous biopsy and concluded that this phenomenon is mainly caused by mechanical factors after surgical excision rather than formalin fixation [14].

## 2. Materials and Methods

### 2.1. Study Design and Setting

We performed a prospective study on formaldehyde-fixed tissue samples at the Pathology Department of Timișoara County Hospital. The primary objective of this study was to evaluate the agreement between ex vivo 22 MHz HFUS measurements and histopathology regarding tumor depth, maximal tumor diameter and deep surgical margins using Bland–Altman methodology.

### 2.2. Participants and Eligibility Criteria

As the study was prospective and the ultrasound operator was blinded to the diagnosis, we included consecutive skin biopsies that mentioned a clinical suspicion of skin tumors on the surgical report. The clinical diagnosis that accompanied the excised tissue ranged from nevi, BCC, melanoma, lipoma, etc. The study represents consecutive cases over 3 months (November 2021–January 2022). A total of 77 consecutive formaldehyde-fixed skin biopsies were systematically scanned with ultrasound, and the images were saved. Of these, 24 biopsies were identified as basal cell carcinomas. A total of 5 ultrasound recordings were not obtained due to technical difficulties, and 19 biopsies from 17 patients were included in the final analysis.

### 2.3. Study Procedures and Data Collection

Our study had 3 separate stages. 1. Consecutive skin biopsy specimens were examined after 6–8 h of formalin fixation. An ultrasound scan of the entire surface along both the long and short axes was recorded on the ultrasound software suite. Before each ultrasound scan there was a short discussion about how the specimen would be sectioned by the pathologist, and that guided the selection of the ultrasound planes. 2. After the results of the pathology reports became available, two researchers independently analyzed the ultrasound data (V.-T.P.) and histopathology reports (B.N.) and recorded their findings in Excel tables. 3. The third and last stage, after completion and verification of all assessments, was to unblind the dataset and connect the measurements using study identifiers.

### 2.4. Ultrasound Examination

We used a 22 MHz mechanical sweeping linear probe, Dub Skin Scanner 75, Taberna Pro Medicum, Germany. The images obtained were measured within the software suite of the ultrasound device. All pathology skin samples were scanned with ultrasound before orientation and paraffin processing. Care was taken to minimize the duration of the exam and the handling of the formaldehyde-fixed tissue. Tissue samples were placed between two layers of ultrasound gel (an initial layer placed on the examination table and a second layer of ultrasound gel applied over the sample). The ultrasound probe was oriented along the long axis, the short axis, and then we performed a sweep of the lesion, noting any particularities. For ideal results, we applied minimal pressure on the ultrasound gel to ensure the tissue sample was not distorted by the pressure of the ultrasound probe. The presence of a clearly visible layer of ultrasound gel on top of the skin sample is a good indicator that there is no excessive pressure being applied (excessive pressure would force the ultrasound gel to the side and leave the probe in direct contact with the tissue).

In cases where the tissue sample was circular or was very small, the probe was swept perpendicularly or over the entire surface.

**Figure 1:**
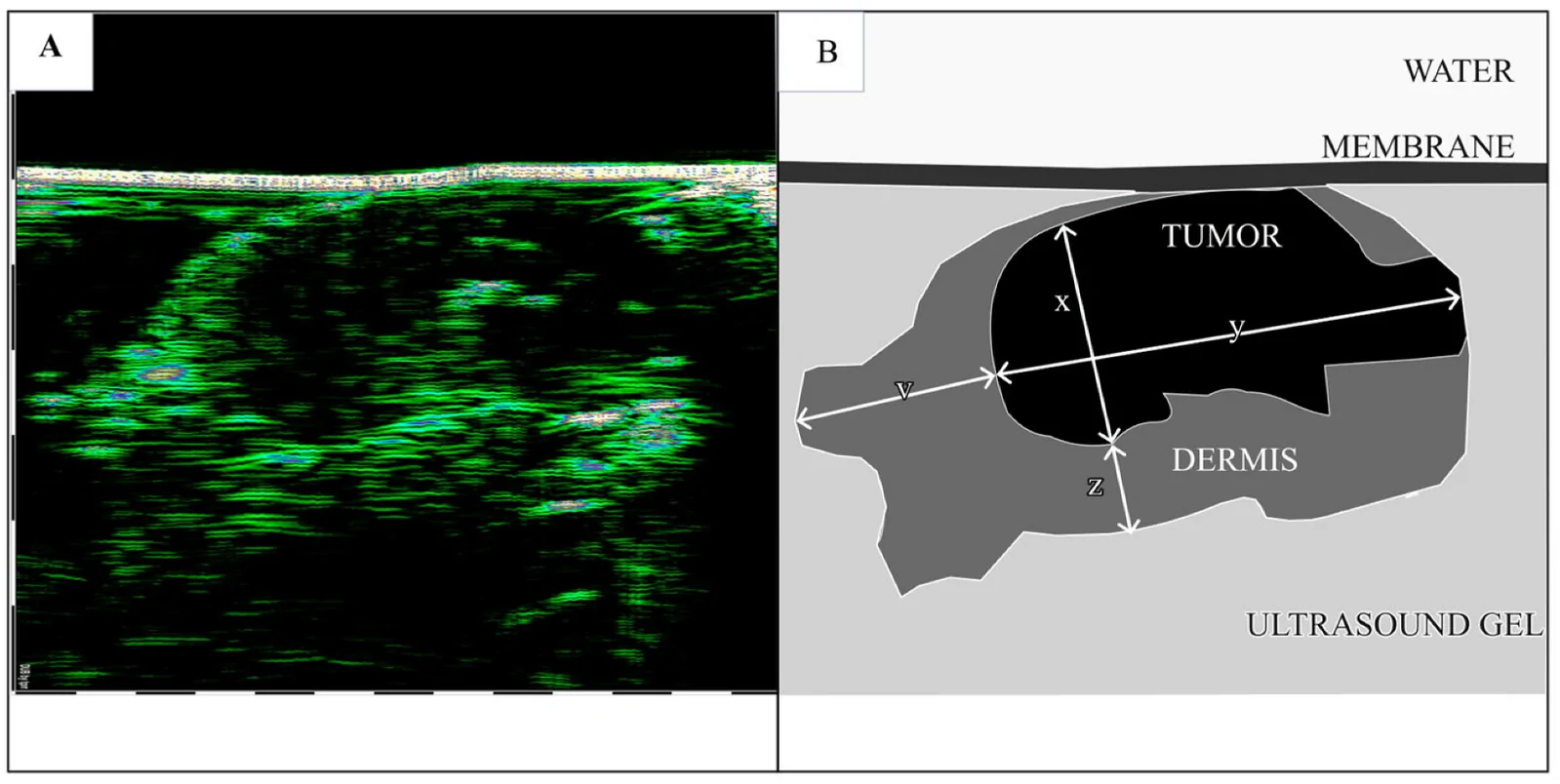
(**A**) The image obtained from a 22 MHz ultrasound of a skin biopsy. Note the black and white lines on the edge of the image representing millimeters. (**B**) The main elements of the ultrasonic image are summarized: x—depth of invasion—the depth of the tumor; y—tumor size (greatest dimension); z—the distance between the tumor and the edge of the sample, also named deep margin or tumor free deep limit of excision; v—the distance between the tumor and the lateral border of the skin sample, also named lateral margin.

The variables measured with ultrasound were chosen to correspond to the ones used in histopathology reports (Figure 1):

1. Depth of invasion (alternative terms: vertical thickness, tumor depth, modified Breslow);
2. Deep margin (tumor free deep limit of excision);
3. Tumor size (alternative terms: horizontal tumor thickness, tumor diameter).

The ultrasound measurements were performed by one author (V.-T.P.), who has over 10 years of experience using HFUS in dermatology. In all cases, the basal cell carcinoma presented as a hypoechoic region relative to the surrounding dermis. The abrupt change in echogenicity was interpreted as the tumor edge. If the hypoechoic region did not end but extended to the edge of the specimen (Figure 1A, right lateral margin), the corresponding margin was recorded as positive. Maximum tumor depth was assessed as the longest line that could be drawn within the tumor perpendicular to the virtual axis of the biopsy specimen (a line running parallel to the physiological epidermis, not the portion raised by the tumor). The deep margin was measured by extending the line used to measure tumor depth to the deep portion of the biopsy specimen (Figure 1B, line z). Finally, maximum diameter was defined simply as the longest line that could be drawn within the tumor. All measurements were made using the dedicated software of the ultrasound device.

### 2.5. Histopathological Examination

After ultrasound scanning, the tissue was returned to the formaldehyde solution, and routine processing followed, including paraffin embedding and hematoxylin and eosin staining. In the case of BCC, the pathology department routinely records maximum tumor depth, the deep margin free from tumor and lateral margins, in both long and short axes. Not all measurements were complete, and where there was missing data, analysis was performed only on the samples that had measurements pertaining to the variable being studied.

### 2.6. Statistical Analysis

A Bland–Altman analysis was considered the most appropriate test to compare the two techniques of measuring tumor size. It can detect bias in measurement (measurement that can become progressively overestimated in larger tumors) and reveals the limits of agreement as defined by the mean difference ±1.96 standard deviations [15,16].

### 2.7. Diagnostic Accuracy Study

Because of the prospective and blinded nature of the study, many lesions were recorded with ultrasound along the long and short axes and then only the ones identified by pathology to be basal cell carcinomas were selected for extensive measurements on the recorded ultrasound images. A participant flow diagram was prepared in accordance with the STARD (“Standards for Reporting Diagnostic accuracy studies”) 2015 guideline [17]. Receiver operating characteristic curve analysis was performed to assess the ability of HFUS to discriminate histopathological tumor depth of over 2 mm. An exploratory hypothesis-generating ultrasound cutoff point was identified within our small dataset using Youden’s index. Sensitivity and specificity were reported for the exploratory ultrasound cutoff.

## 3. Results

### 3.1. Study Population

Our study population consisted of 17 patients who contributed 19 distinct lesions. Sex distribution was predominantly male (76.5% vs. 23.5%) with a median age of 73 years (interquartile range 62–77 years). Lesions were excised mainly from the head and neck area (84.2%) and histologically comprised mainly of nodular (42.1%) and infiltrative (36.8%) subtypes (Table 1). Two infiltrative lesions presented areas of nodular basal cell carcinoma. All lesions were fully excised.

### 3.2. Bland–Altman Analysis of Measurements

The results of the Bland–Altman analysis are presented in Table 2. The arithmetic mean of the differences ranged from +0.21 mm (CI –0.69 to 1.12 mm) for tumor depth measurement to −1.38 mm (CI −5.43 to 2.66) in the case of tumor size. Therefore, there is no evidence of a significant overall average bias.

On the other hand, the limits of agreement are wide, as, for example, in the case of tumor depth, the upper and lower limits of agreement are 3.77 mm (95% confidence interval 2.20 to 5.35 mm) and −3.35 mm (CI −4.92 to −1.78 mm), respectively.

#### 3.2.1. Depth of Invasion

**Figure 2:**
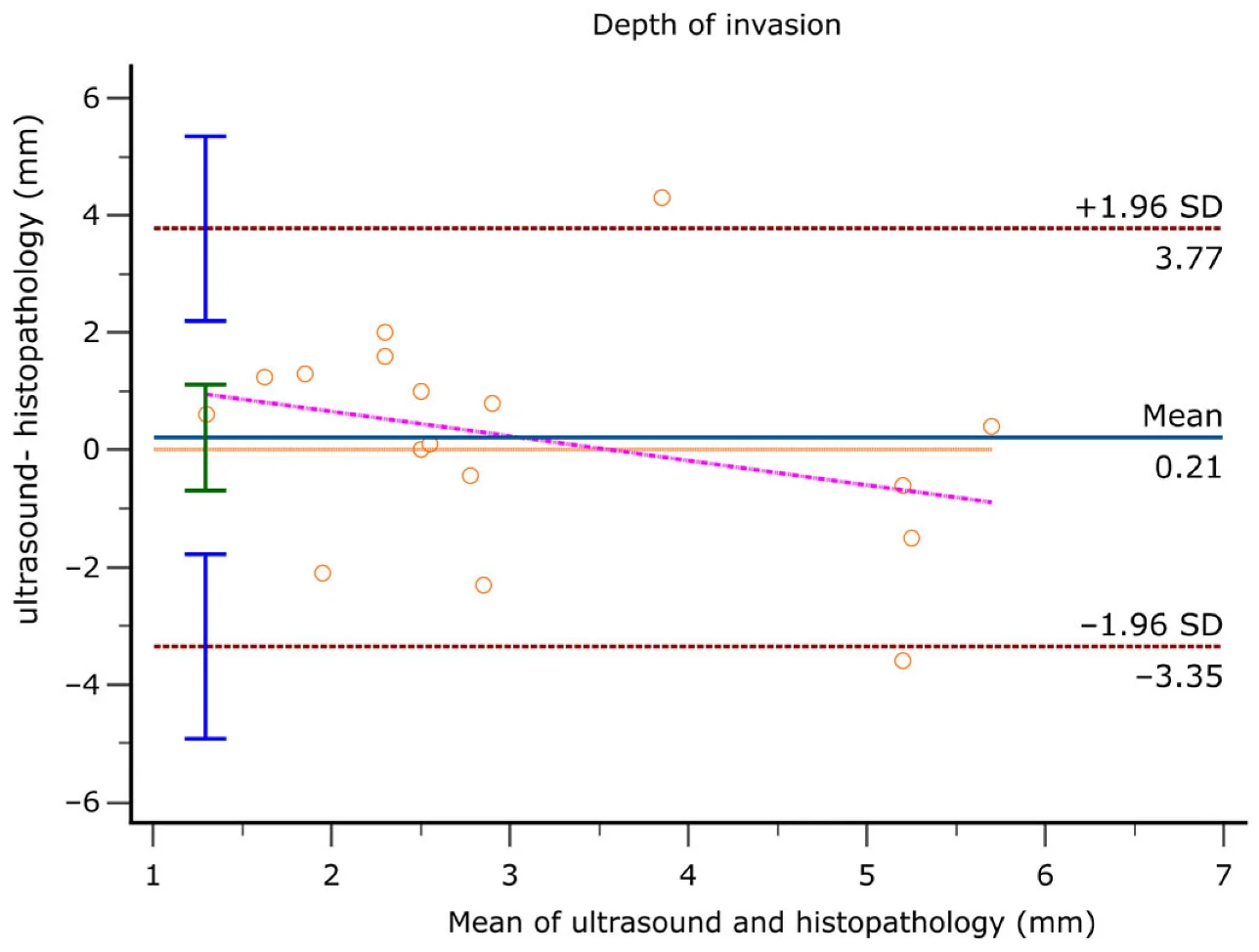
Bland–Altman plot of ultrasound minus histopathology measurements of depth of invasion against their mean (orange circles). Horizontal lines indicate perfect agreement (orange), mean difference (blue), and 95% limits of agreement (red dashed). Green and blue brackets show the respective 95% confidence intervals for the mean difference and limits of agreement. The pink line indicates the fitted linear trend.

Graphical representation of the Bland–Altman analysis of the tumor depth measurements complete with a regression line (Figure 2). Despite the slight positive intercept on the y-axis (+1.4 mm) and a downward slope (−0.42 mm), neither value was statistically significant (*p* = 0.17 and *p* = 0.2, respectively).

#### 3.2.2. Deep Margin

**Figure 3:**
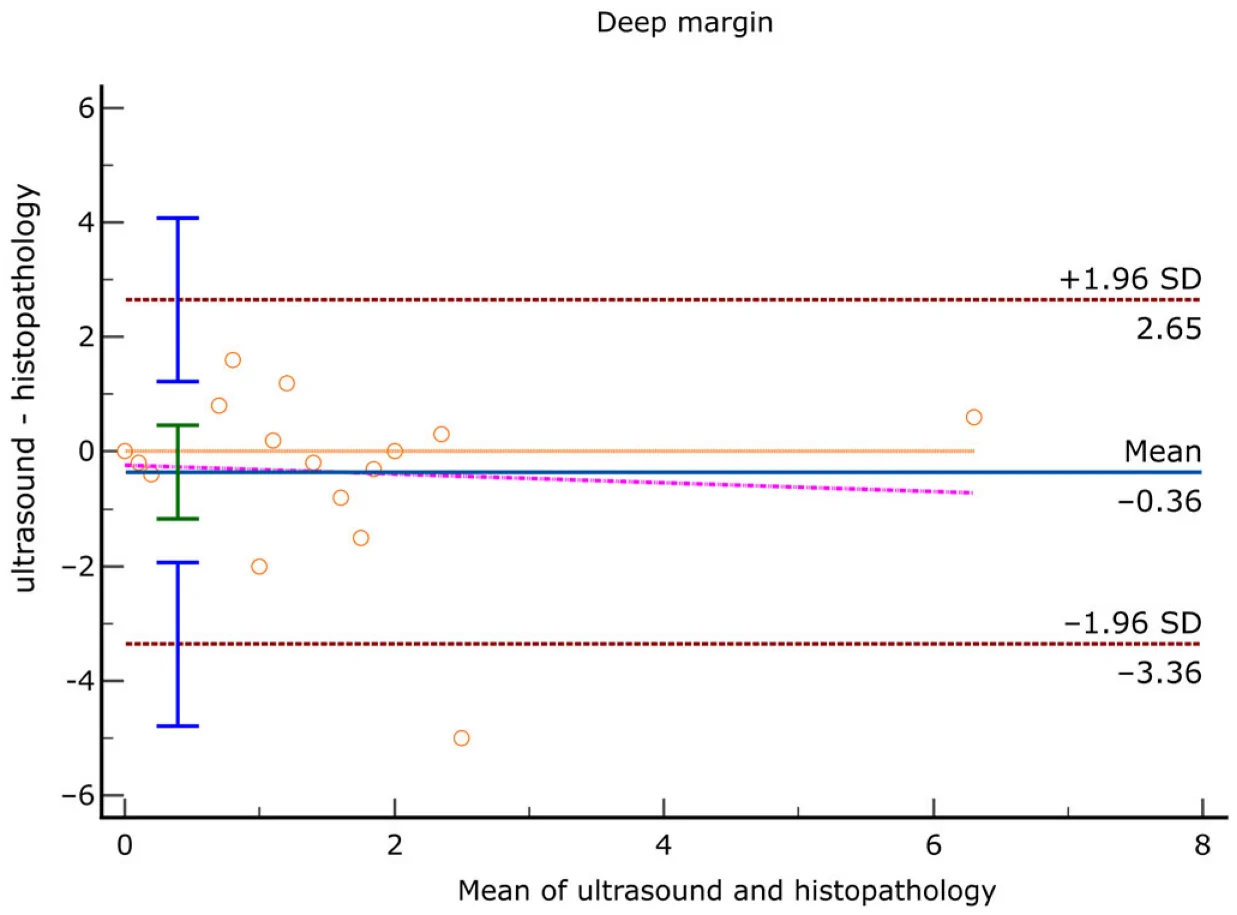
Bland–Altman plot of ultrasound minus histopathology measurements of deep margin against their mean (orange circles). Horizontal lines indicate perfect agreement (orange), mean difference (blue), and 95% limits of agreement (red dashed). Green and blue brackets show the respective 95% confidence intervals for the mean difference and limits of agreement. The pink line indicates the fitted linear trend.

Graphical representation of the Bland–Altman analysis of the deep margin measurements complete with a regression line (Figure 3). The regression line intercepts the y-axis at a value of −0.2 mm and shows a small downward slope (−0.08). Both values do not reach statistical significance (*p* = 0.69 and *p* = 0.78). There is therefore no evidence of systematic bias.

#### 3.2.3. Tumor Size

**Figure 4:**
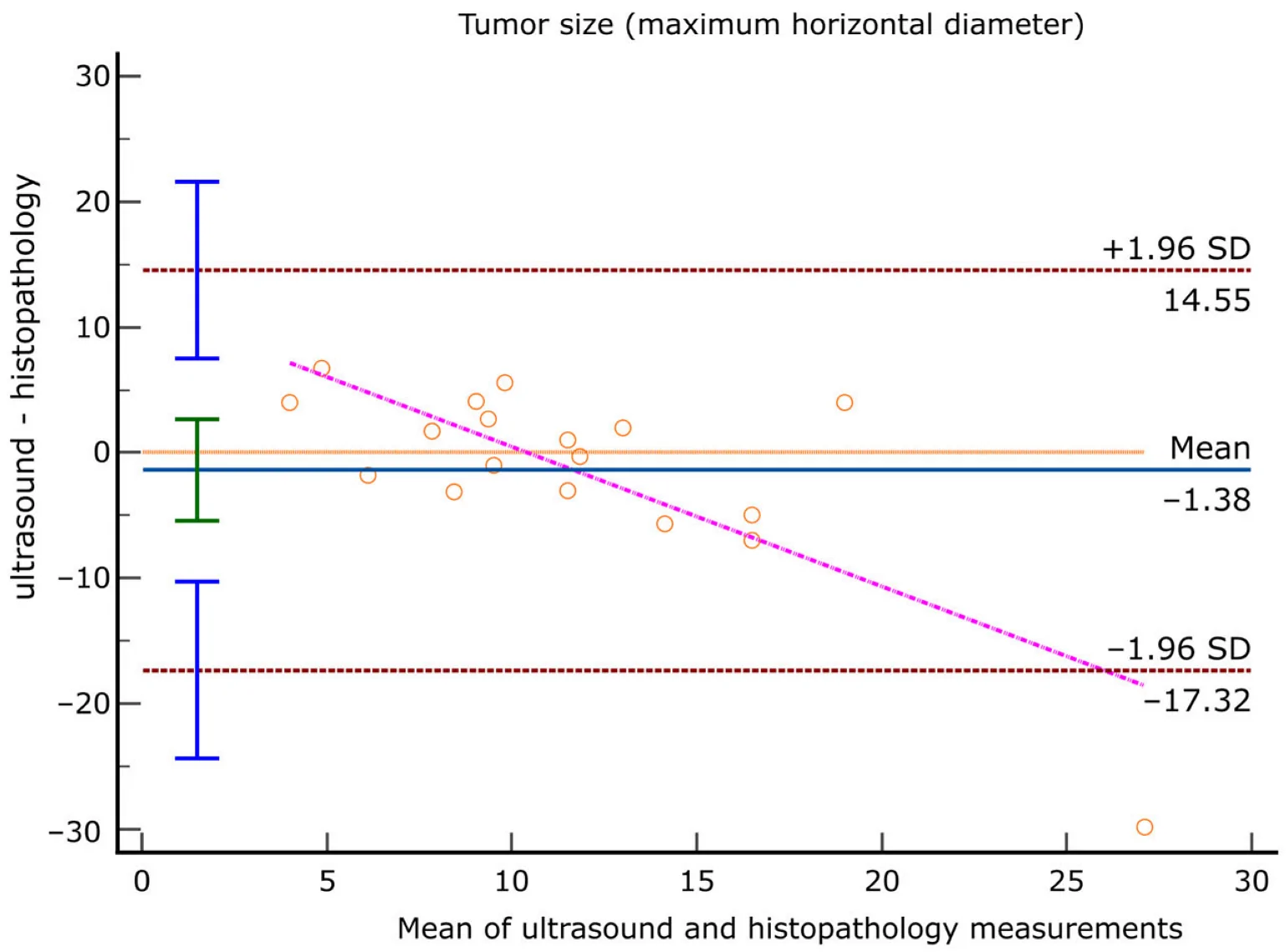
Bland–Altman plot of ultrasound minus histopathology measurements of tumor size against their mean (orange circles). Horizontal lines indicate perfect agreement (orange), mean difference (blue), and 95% limits of agreement (red dashed). Green and blue brackets show the respective 95% confidence intervals for the mean difference and limits of agreement. The pink line indicates the fitted linear trend.

Figure 4 presents the Bland–Altman analysis of the tumor size measurement complete with a regression line (Figure 4). The regression line intercepts the y-axis at a value of 11.6 mm (*p* = 0.0015). The slope value is −1.11 (*p* = 0.0002); therefore, there is proportional bias. There was no evidence of systematic bias. The limits of agreement are wide (−17.32 to 14.55 mm).

### 3.3. Exploratory Analysis of Diagnostic Accuracy

**Figure 5:**
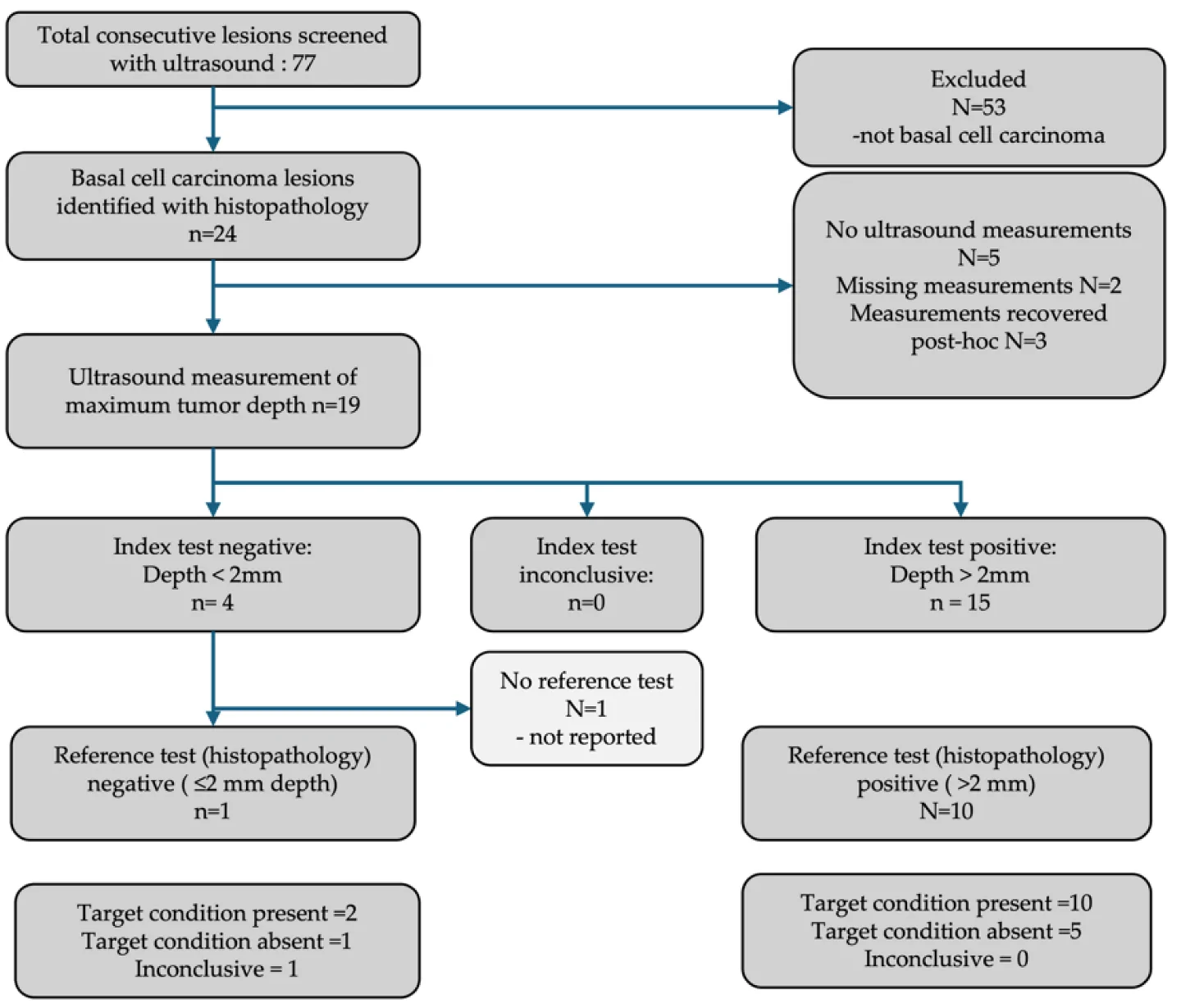
Biopsy flow diagram for ROC analysis.

Out of a total of 77 individual lesions scanned with ultrasound, only 24 were identified as being BCC. Five sets of ultrasound measurements were not available for the blinded analysis (two missing datasets with files possibly corrupted and impossible to open, and three were recovered post hoc and were not included to retain the blinded nature of the study). One small lesion, with ample excision margins, did not have tumor depth in the report. (Figure 5).

**Figure 6:**
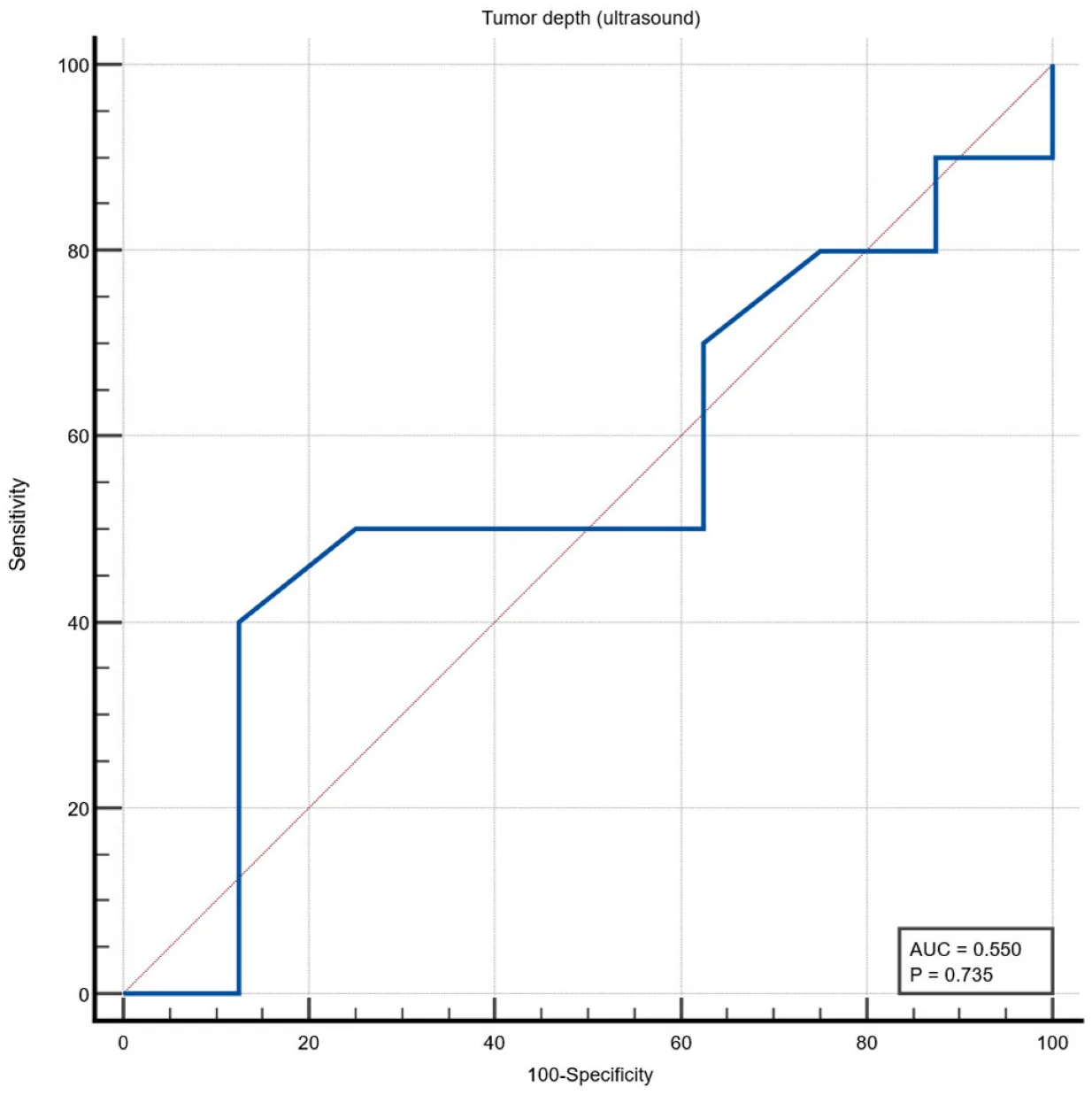
Exploratory receiver operating characteristic (ROC) curve for HFUS-measured tumor depth used to identify tumor with a histopathological depth of >2 mm. AUC—area under the curve.

Exploratory receiver operating characteristic (ROC) curve analysis of the accuracy of HFUS in correctly identifying BCC with a depth of more than 2 mm yielded an area under the curve (AUC) of 0.55 (95% confidence interval 0.30 to 0.78), *p* = 0.74. A sensitivity of 40% and specificity of 87.5% were obtained by the Youden index at a value of >3.3 mm on ultrasound (Figure 6).

## 4. Discussion

In this small pilot study, ex vivo ultrasound did not prove interchangeable with histopathology. For all variables measured (tumor depth, deep margin, maximum diameter), the 95% confidence interval of the mean difference in the Bland–Altman analysis included zero, providing no evidence of an overall mean bias. The mean bias of the tumor depth measurements is +0.21 mm, which is in line with the recent article by Popescu et al. that found a mean bias of (−0.07) [13]. Our limits of agreement ranged from −3.35 to +3.77 in the case of tumor depth. These limits are large by clinical standards. A recent clinical guideline (S2k) offers a 2 mm depth as a possible cutoff point when choosing treatment in BCC. The same guideline mentions that there is no clear consensus regarding the significance of tumor depth. The NCCN BCC guideline [18] does not mention tumor depth but considers a diameter of over 6 mm in high risk areas and 10 mm in moderate risk areas as a marker of aggressive BCC. In our study population, the limits of agreement for maximum diameter ranged from −17.32 to +14.55. These limits are too wide to permit confident classification into these categories. Rather than discouraging evaluation with cutaneous ultrasound, these results underline the importance of well-designed studies on prospective consecutive cohorts, that have the possibility of signaling areas that can be improved in how we obtain and report ultrasound data in HFUS.

The exploratory diagnostic accuracy analysis for the tumor depth did not reliably discriminate tumors that had a depth of over 2 mm on histopathology. The area under the ROC curve was modest. The analysis of the Youden index derived within this small dataset provides an exploratory cutoff of >3.3 mm on ultrasound at which point the sensitivity is 40% and specificity is 87.5% for a histological cutoff of >2 mm tumor depth. This is to be expected, as the previously stated limits of agreement for tumor depth suggest a possible over or underestimation by over 3 mm at an individual lesion level. It is plausible that a choice of a 50 MHz device could be better suited to the analysis of BCC. Ideally, a system with variable frequency could allow the operator to optimize the image acquisition for each patient.

One of the main strengths of this study was a rigorous methodology. The study was prospective, the cases were consecutive, we adhered to the STARD guideline for reporting diagnostic accuracy tests and transparently reported missing cases. Bland–Altman analysis is well suited to comparing methods of measurement, especially if one of them is the gold standard [15,16]. The main utility in our study was the ability of the method to discriminate between a small overall mean bias that contrasted with clinically wide limits of agreement.

Performing HFUS and interpreting the data can be influenced by operator experience, hardware selection, technique and tissue-specific factors. A potential limitation in our study was that only one researcher interpreted the ultrasound data. It would be interesting to compare measurements made by multiple operators, to assess the reproducibility of ultrasound measurement. The selection of the ultrasound probe is another limitation because the difference in size, shape, grip and ease of use can influence image acquisition. Software also plays an important role because the availability of special modes such as panoramic image acquisition or the ability to conduct measurements on saved files can differ between manufacturers. Technique plays a role as pressure distorts the skin. Ideally, a thick layer of ultrasound gel should clearly be visible on top of the skin. Lastly, the shrinkage of the skin specimen after biopsy is a well-known factor. Dauendorffer et al. reported different percentages for length versus width (16% versus 18%) and noted that the shrinkage effect is more pronounced with larger sizes or for skin removed from the limbs. Most importantly, the study found that shrinkage manifests after excision and before formalin fixation [14]. These findings do not, however, account for possible changes during paraffin wax embedding, sectioning and slide preparation. This is a potential source of variability in the measurements, and it would be an interesting area of research in the future. Ideally, a morphological study could follow a basal cell carcinoma from in vivo to ex vivo (intraoperative and after formalin fixation) and then one could even attempt the examination of paraffin wax blocks with the aid of ultrasound or optical coherence tomography. The absence of in vivo measurements is a limitation of our study.

## 5. Conclusions

Ex vivo 22 MHz HFUS showed insufficient agreement with histopathology to be considered interchangeable for quantitative morphological assessment of BCC. Further studies should investigate the potential role of HFUS in preoperative and intraoperative assessment of BCC and determine whether clinically useful thresholds can be established. To our knowledge, this is the first prospective study to rigorously evaluate the limits of agreement between ex vivo high-frequency ultrasound (HFUS) and histopathology using a Bland–Altman approach, thereby establishing a methodological framework for future studies in dermato-oncology.

## Figures and Tables

**Table 1:** Demographics and clinical characteristics.

Characteristic	Value (%)
No. of patients	17
Sex	
Male	13 (76.5)
Female	4 (23.5)
Age *	73 (62–77)
No. of distinct lesions	19
Location of BCC:	
Head and neck	16 (84.2)
Trunk	2 (10.5)
Upper and lower limbs	1 (5.3)
Histological subtype:	
Nodular	8 (42.1)
Infiltrative	7 (36.8)
Superficial	4 (21.1)

* Age is reported as median (interquartile range).

**Table 2:** Bland–Altman analysis.

Measurement	Depth of Invasion	Deep Margin	Tumor Size
Sample Size	18	16	18
Arithmetic Mean	0.2117	−0.36	−1.38
95% Confidence Interval	−0.69 to 1.12	−1.17 to 0.46	−5.43 to 2.66
Lower Limit	−3.35	−3.36	−17.32
95% Confidence Interval Lower	−4.92 to −1.78	−4.79 to −1.94	−24.36 to −10.27
Upper Limit	3.77	2.65	14.55
95% Confidence Interval Upper	2.20 to 5.35	1.22 to 4.08	7.51 to 21.59

## Data Availability

The data presented in this study are available on request from the corresponding author due to privacy.

## Abbreviations

The following abbreviations are used in this manuscript:

BCC	Basal cell carcinoma
HFUS	High-frequency ultrasound of the skin
OCT	Optical coherence tomography
RCM	Reflectance confocal microscopy
ROC	Receiver operating characteristic
AUC	Area under the curve
STARD	Standards for Reporting Diagnostic accuracy studies
